# Supplementary material for: Economic Evaluation of Hepatitis C Treatment Extension to Acute Infection and Early-Stage Fibrosis Among Patients Who Inject Drugs in Developing Countries: A Case of China
Source: Int J Environ Res Public Health. 2020 Jan 28;17(3):800. doi: 10.3390/ijerph17030800 (PMC7037788; doi:10.3390/ijerph17030800)
Supplement: Supplementary file 1 [file ijerph-17-00800-s001.zip › Supplement materials.docx]

**Economic evaluation of hepatitis C treatment extension to acute infection and early-stage fibrosis among patients who inject drugs in developing countries: a case of China**

**Table of contents**

**Table S1…………………………………………………………………………………………………..2**

**Figure S1…………………………………………………………………………………………………4**

**Figure S2…………………………………………………………………………………………………5**

**Figure S3…………………………………………………………………………………………………6**

**Figure S4…………………………………………………………………………………………………7**

**Figure S5…………………………………………………………………………………………………8**

**Figure S6…………………………………………………………………………………………………9**

**Table S1. Baseline model input variables for Markov model assessment**

| **Cohort characteristics** |  | |  | |  | |  |  | Reference |  |
| --- | --- | --- | --- | --- | --- | --- | --- | --- | --- | --- |
| Cohort size |  |  | |  | | 1,000 | | | | |
| Starting condition | Acute and Chronic (F0-F4) | | | | | | | |  |  |
| Mean age of infection | 20.7 (15.8-23.0) | | | | | | | | ([1](#_ENREF_1)) |  |
| Annual discount | 5% (3%-5%) | | | | | | |  |  |  |
|  | Base case | | 95%CI | | Distribution | | Parameter 1^a^ | Parameter 2^b^ | Reference |  |
| **Transition probabilities** |  | |  | |  | |  |  |  |  |
| F0 to F1 | 0.128 | | 0.080-0.176 | | Beta | | 23.69 | 161.41 | ([1](#_ENREF_1)) |  |
| F1 to F2 | 0.059 | | 0.035-0.082 | | Beta | | 21.79 | 347.49 | ([1](#_ENREF_1)) |  |
| F2 to F3 | 0.078 | | 0.056-0.100 | | Beta | | 44.45 | 525.37 | ([1](#_ENREF_1)) |  |
| F3 to F4 | 0.116 | | 0.070-0.161 | | Beta | | 21.48 | 163.69 | ([1](#_ENREF_1)) |  |
| F4 to DC | 0.039 | | 0.035-0.043 | | Beta | | 350.85 | 8645.24 | ([2](#_ENREF_2)) |  |
| F4 to HCC | 0.025 | | 0.022-0.028 | | Beta | | 259.95 | 10138.11 | ([2](#_ENREF_2)) |  |
| DC to HCC | 0.068 | | 0.048-0.088 | | Beta | | 41.32 | 566.35 | ([3](#_ENREF_3), [4](#_ENREF_4)) |  |
| DC to LT | 0.008 | | 0.006-0.01 | | Beta | | 61.01 | 7565.82 | ([5](#_ENREF_5)) |  |
| HCC to LT | 0.008 | | 0.006-0.01 | | Beta | | 61.01 | 7565.82 | ([5](#_ENREF_5)) |  |
| Decrement of F4_SVR-DC | 0.913 | | 0.730-1.000 | | Beta | | 7.41 | 0.71 | ([6](#_ENREF_6)) |  |
| Decrement of F4_SVR-HCC | 0.764 | | 0.611-0.917 | | Beta | | 21.84 | 6.75 | ([6](#_ENREF_6)) |  |
| **Fibrosis regression Post-SVR** | | |  | |  | |  |  | ([7](#_ENREF_7)) |  |
| F1_SVR to F0_SVR | 0.35 | | 0.17-0.52 | | Beta | | 9.09 | 16.88 |  |  |
| F2_SVR to F0_SVR | 0.12 | | 0.06-0.18 | | Beta | | 13.40 | 98.29 |  |  |
| F2_SVR to F1_SVR | 0.58 | | 0.29-0.87 | | Beta | | 5.87 | 4.25 |  |  |
| F3_SVR to F1_SVR | 0.24 | | 0.12-0.36 | | Beta | | 11.44 | 36.22 |  |  |
| F3_SVR to F2_SVR | 0.46 | | 0.23-0.69 | | Beta | | 7.84 | 9.20 |  |  |
| F4_SVR to F1_SVR | 0.09 | | 0.05-0.14 | | Beta | | 17.61 | 178.04 |  |  |
| F4_SVR to F2_SVR | 0.14 | | 0.07-0.21 | | Beta | | 13.08 | 80.32 |  |  |
| F4_SVR to F3_SVR | 0.22 | | 0.11-0.33 | | Beta | | 11.77 | 41.72 |  |  |
| **Reinfection** |  | |  | |  | |  |  |  |  |
| Reinfection rate after clearing virus | 0.190 | | 0.006-0.190 | | Uniform | | 0.006 | 0.19 | ([8](#_ENREF_8), [9](#_ENREF_9)) |  |
| Reclearance proportion within 6 months after reinfection | 0.52 | | 0.33-0.73 | | Beta | | 13.27 | 12.25 | ([10](#_ENREF_10)) |  |
| **Mortality** |  | |  | |  | |  |  |  |  |
| Overdose-related mortality | 0.0065 | | 0.0055-0.0075 | | Beta | | 161.38 | 24665.58 | ([11](#_ENREF_11)) |  |
| DC to death | 0.129 | | 0.103-0.155 | | Beta | | 83.52 | 563.93 | ([4](#_ENREF_4)) |  |
| HCC to death | 0.427 | | 0.342-0.512 | | Beta | | 54.60 | 73.27 | ([4](#_ENREF_4)) |  |
| LT death first year | 0.140 | | 0.126-0.154 | | Beta | | 330.22 | 2028.52 | ([12](#_ENREF_12)) |  |
| LT death second year and beyond | 0.025 | | 0.023-0.027 | | Beta | | 585.69 | 22841.80 | ([12](#_ENREF_12)) |  |
| **Spontaneous clearance rate following acute primary infection** | 0.26 | | 0.22-0.29 | | Beta | | 124.8 | 355.2 | ([13](#_ENREF_13)) |  |
| **Probability of SVR** |  | |  | |  | |  |  |  |  |
| pegIFN for acute infection | 0.71 | | 0.71-0.94 | | Uniform | | 0.71 | 0.94 | ([14](#_ENREF_14)) |  |
| PegIFN+RBV for chronic infection | 0.520 | | 0.340-0.690 | | Beta | | 14.88 | 13.74 | ([15-17](#_ENREF_15)) |  |
| DCV+ASV | 0.898 | | 0.863-0.924 | | Beta | | 257.05 | 29.20 | ([18](#_ENREF_18)) |  |
| **Discontinuation rate of treatment** | | |  | |  | |  |  |  |  |
| PegIFN-based | 0.200 | | 0.087-0.340 | | Beta | | 9.43 | 37.71 | ([15](#_ENREF_15)) |  |
| DCV+ASV | 0.046 | | 0.006-0.050 | | Uniform | | 0.006 | 0.050 | ([19-21](#_ENREF_19)) |  |
| **Costs in 2018 US dollars** |  | |  | |  | |  |  |  |  |
| **Treatment costs** |  | |  | |  | |  |  |  |  |
| PegIFN per week | 168.4 | | 132.8-203.6 | | Gamma | | 87.53 | 0.53 | ([22](#_ENREF_22)) |  |
| PegIFN+RBV per week | 170.9 | | 136.7-205.1 | | Gamma | | 95.99 | 0.56 | ([21](#_ENREF_21)) |  |
| DCV+ASV peer week | 359.5 | | 287.6-431.4 | | Gamma | | 96.06 | 0.27 | ([21](#_ENREF_21)) |  |
| **Treatment monitoring** |  | |  | |  | |  |  |  |  |
| HCV RNA test | 30 | | 22.5-37.5 | | Gamma | | 97.6 | 2.98 | Local price |  |
| Genotype test | 75 | | 56-94 | | Gamma | | 59.86 | 0.80 | Local price |  |
| Annual managing F0-F3 HCV disease^#^ | 851 | | 575-1,126 | | Gamma | | 36.69 | 0.04 | ([23](#_ENREF_23)) |  |
| Annual managing F4 HCV disease^#^ | 2,421 | | 858-3,984 | | Gamma | | 9.22 | 0.004 | ([23](#_ENREF_23)) |  |
| Annual managing DC disease^#^ | 5,392 | | 3,276-7,507 | | Gamma | | 24.95 | 0.004 | ([23](#_ENREF_23)) |  |
| Annual managing HCC disease^#^ | 11,380 | | 8,184-14,577 | | Gamma | | 48.70 | 0.004 | ([23](#_ENREF_23)) |  |
| Annual managing LT in 1st year^*^ | 54,920 | | 39,682-79,363 | | Gamma | | 34.58 | 5.81 | ([24](#_ENREF_24)) |  |
| Annual managing LT in subsequent year^*^ | 8,730 | | 7,936-9,757 | | Gamma | | 362.66 | 0.04 | ([24](#_ENREF_24)) |  |
| **Utility values** |  | |  | |  | |  |  |  |  |
| F0-F1 | 0.98 | | 0.92-1.00 | | Beta | | 91.20 | 3.80 | ([25](#_ENREF_25), [26](#_ENREF_26)) |  |
| F2 | 0.92 | | 0.72-1.00 | | Beta | | 19.43 | 3.16 | ([26](#_ENREF_26)) |  |
| F3 | 0.79 | | 0.77-0.81 | | Beta | | 1309.82 | 348.18 | ([27](#_ENREF_27)) |  |
| F4 | 0.76 | | 0.70-0.79 | | Beta | | 267.76 | 91.65 | ([27](#_ENREF_27)) |  |
| F0_SVR-F1_SVR | 1.00 | | 0.98-1.00 | | Beta | | 375.53 | 3.793 | ([25](#_ENREF_25)) |  |
| F2_SVR | 0.933 | | 0.92-1.00 | | Beta | | 87.55 | 3.65 | ([25](#_ENREF_25)) |  |
| F3_SVR | 0.86 | | 0.82-0.90 | | Beta | | 247.75 | 40.33 | ([28](#_ENREF_28)) |  |
| F4_SVR | 0.83 | | 0.79-0.87 | | Beta | | 280.36 | 57.42 | ([28](#_ENREF_28)) |  |
| DC | 0.69 | | 0.44-0.69 | | Beta | | 33.58 | 25.85 | ([27](#_ENREF_27)) |  |
| HCC | 0.67 | | 0.60-0.72 | | Beta | | 157.41 | 77.53 | ([27](#_ENREF_27)) |  |
| LT in 1^st^ year | 0.50 | | 0.40-0.69 | | Beta | | 24.15 | 20.16 | ([27](#_ENREF_27)) |  |
| LT in subsequent year | 0.77 | | 0.57-0.77 | | Beta | | 56.24 | 27.70 | ([27](#_ENREF_27)) |  |
| **Disutility values** |  | |  | |  | |  |  |  |  |
| PegIFN-based treatment | 0.10 | | 0.04-0.16 | | Beta | | 9.50 | 85.54 | ([22](#_ENREF_22)) |  |
| Oral regimen treatment | 0.05 | | 0-0.10 | | Beta | | 3.60 | 68.39 | ([22](#_ENREF_22)) |  |

^a^ Parameter 1 corresponds to α parameter for beta distribution and *k*(shape) parameter for gamma distribution.

^b^ Parameter 2 corresponds to β parameter for beta distribution and *θ*(shape) parameter for gamma distribution.

^#^ Converted from 2014 USD$(2014US $1=6.14 CNY) to 2018 USD$(2018 US $1=6.62 CNY).

^*^ Converted from 2009 USD$(2009US $1=6.83 CNY) to 2018 USD$(2018 US $1=6.62 CNY).


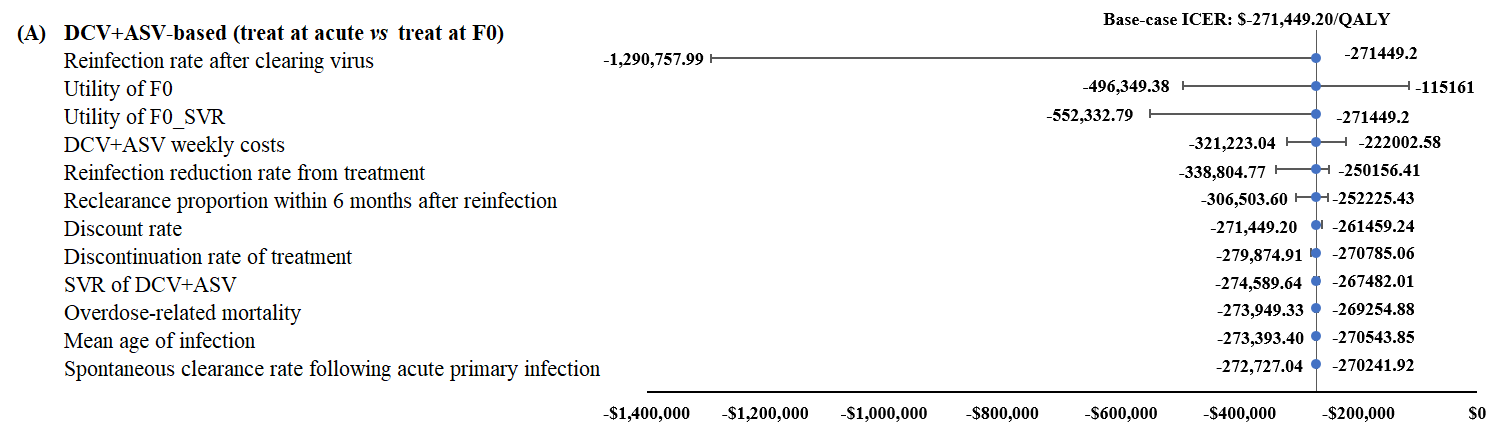


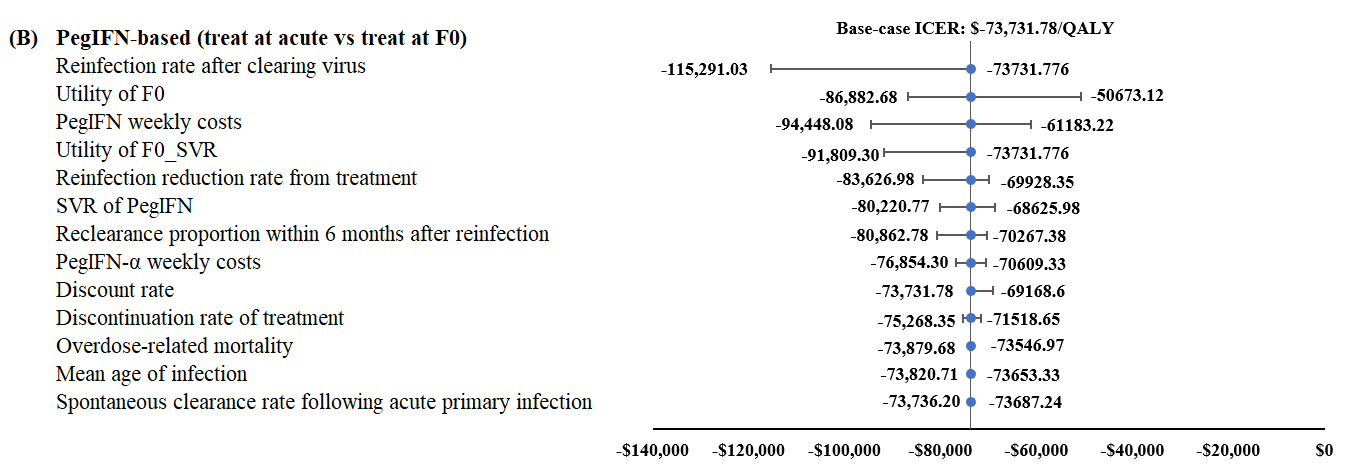


**Figure S1.** One-way sensitivity analysis of incremental cost-effectiveness ratios (ICERs) for treating acute infection *vs* deferring treatment until stage F0 among PWID.


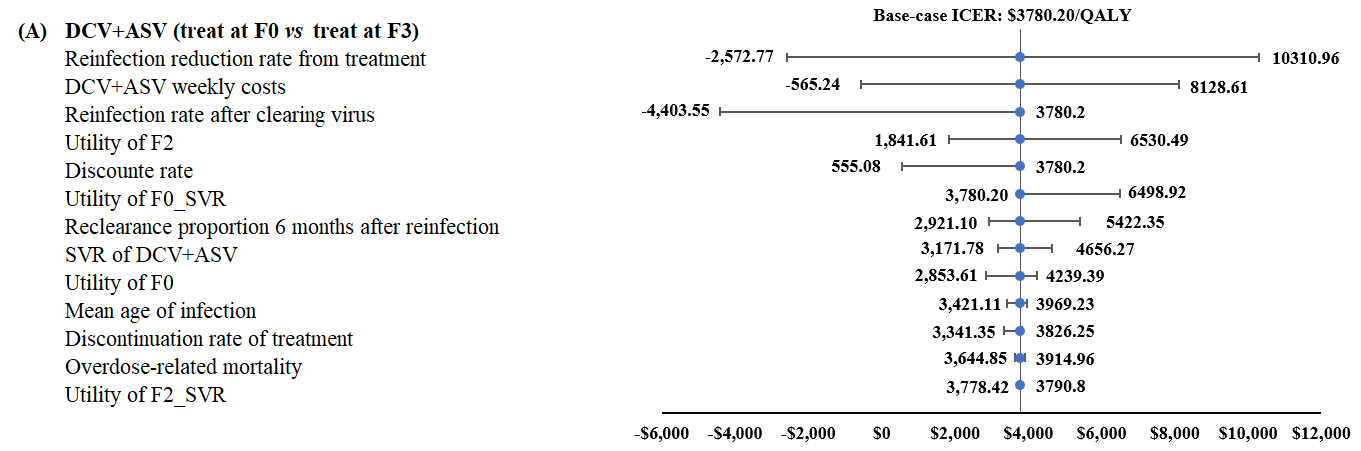


**
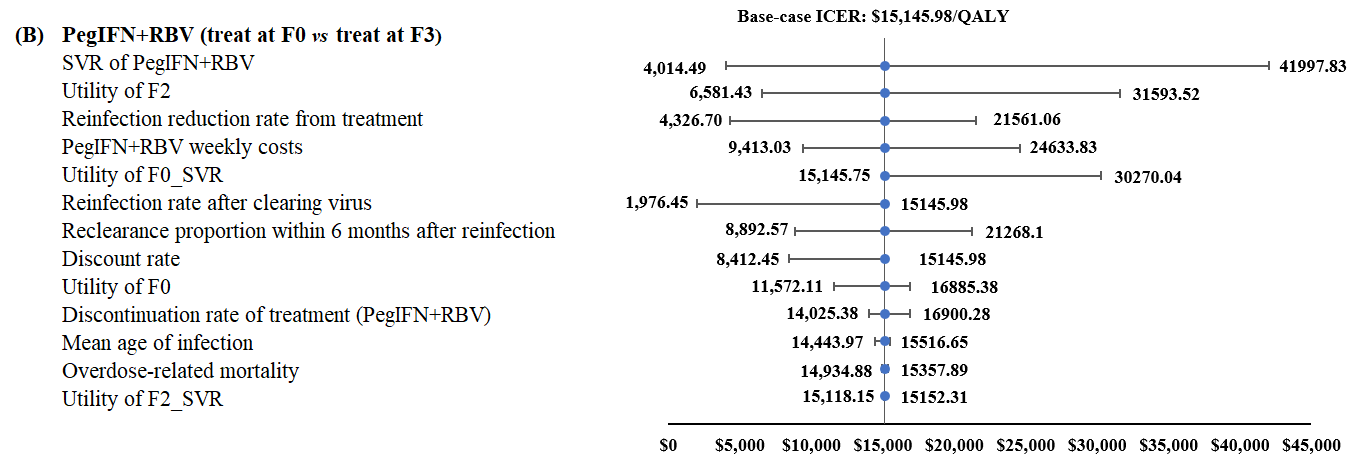
**

**Figure S2.** One-way sensitivity analysis of incremental cost-effectiveness ratios (ICERs) for early treatment at stage F0 vs delayed treatment at F3 among PWID.

**(A)**

**(B)**

**(C)**

**Figure S3.** The impact of SVR of PR (A), utility of F2 (B), utility of F0_SVR (C) on ICER of treatment at F0 *vs* at F3 using PegIFN-based regimen.

**Figure S4**. Cost-effectiveness acceptability curves for treatment at acute compared with at F0 under DCV+ASV or PegIFN-based regimen.

**1 GDP**

**3 GDP**

**Figure S5**. Cost-effectiveness acceptability curves for treatment at F0 compared with at F3 under DCV+ASV or PegIFN-based regimen.

**Reference**

1. Smith DJ, Combellick J, Jordan AE, et al. Hepatitis C virus (HCV) disease progression in people who inject drugs (PWID): A systematic review and meta-analysis. The International journal on drug policy. 2015; 26: 911-21.

2. Rein DB, Wittenborn JS, Smith BD, et al. The cost-effectiveness, health benefits, and financial costs of new antiviral treatments for hepatitis C virus. Clinical infectious diseases : an official publication of the Infectious Diseases Society of America. 2015; 61: 157-68.

3. Planas R, Balleste B, Alvarez MA, et al. Natural history of decompensated hepatitis C virus-related cirrhosis. A study of 200 patients. Journal of hepatology. 2004; 40: 823-30.

4. Fattovich G, Giustina G, Degos F, et al. Morbidity and mortality in compensated cirrhosis type C: a retrospective follow-up study of 384 patients. Gastroenterology. 1997; 112: 463-72.

5. Lo AO, Chan HL, Wong VW, et al. Cost-effectiveness of the highly effective direct-acting antivirals in the treatment of chronic hepatitis C in Hong Kong. Journal of gastroenterology and hepatology. 2017; 32: 1071-78.

6. Chen P, Ma A, Liu Q. Cost-Effectiveness of Elbasvir/Grazoprevir Versus Daclatasvir Plus Asunaprevir in Patients with Chronic Hepatitis C Virus Genotype 1b Infection in China. Clinical drug investigation. 2018; 38: 1031-39.

7. Chahal HS, Marseille EA, Tice JA, et al. Cost-effectiveness of Early Treatment of Hepatitis C Virus Genotype 1 by Stage of Liver Fibrosis in a US Treatment-Naive Population. JAMA Intern Med. 2016; 176: 65-73.

8. Grebely J, Knight E, Ngai T, et al. Reinfection with hepatitis C virus following sustained virological response in injection drug users. Journal of gastroenterology and hepatology. 2010; 25: 1281-4.

9. Martinello M, Dore GJ, Matthews GV, et al. Strategies to Reduce Hepatitis C Virus Reinfection in People Who Inject Drugs. Infectious disease clinics of North America. 2018; 32: 371-93.

10. Sacks-Davis R, Grebely J, Dore GJ, et al. Hepatitis C Virus Reinfection and Spontaneous Clearance of Reinfection--the InC3 Study. The Journal of infectious diseases. 2015; 212: 1407-19.

11. Degenhardt L, Bucello C, Mathers B, et al. Mortality among regular or dependent users of heroin and other opioids: a systematic review and meta-analysis of cohort studies. Addiction (Abingdon, England). 2011; 106: 32-51.

12. He T, Li K, Roberts MS, et al. Prevention of Hepatitis C by Screening and Treatment in U.S. Prisons. Annals of internal medicine. 2016; 164: 84-92.

13. Micallef JM, Kaldor JM, Dore GJ. Spontaneous viral clearance following acute hepatitis C infection: a systematic review of longitudinal studies. Journal of viral hepatitis. 2006; 13: 34-41.

14. Hullegie SJ, Arends JE, Rijnders BJ, et al. Current knowledge and future perspectives on acute hepatitis C infection. Clinical microbiology and infection : the official publication of the European Society of Clinical Microbiology and Infectious Diseases. 2015; 21: 797 e9-97 e17.

15. Dimova RB, Zeremski M, Jacobson IM, et al. Determinants of hepatitis C virus treatment completion and efficacy in drug users assessed by meta-analysis. Clinical infectious diseases : an official publication of the Infectious Diseases Society of America. 2013; 56: 806-16.

16. Hilsden RJ, Macphail G, Grebely J, et al. Directly observed pegylated interferon plus self-administered ribavirin for the treatment of hepatitis C virus infection in people actively using drugs: a randomized controlled trial. Clinical infectious diseases : an official publication of the Infectious Diseases Society of America. 2013; 57 Suppl 2: S90-6.

17. Zanini B, Covolo L, Donato F, et al. Effectiveness and tolerability of combination treatment of chronic hepatitis C in illicit drug users: Meta-analysis of prospective studies. Clinical therapeutics. 2010; 32: 2139-59.

18. Wang HL, Lu X, Yang X, et al. Effectiveness and safety of daclatasvir plus asunaprevir for hepatitis C virus genotype 1b: Systematic review and meta-analysis. Journal of gastroenterology and hepatology. 2017; 32: 45-52.

19. Boglione L, Mornese Pinna S, De Nicolo A, et al. Treatment with direct-acting antiviral agents of hepatitis C virus infection in injecting drug users: A prospective study. Journal of viral hepatitis. 2017; 24: 850-57.

20. Grebely J, Dalgard O, Conway B, et al. Efficacy and safety of sofosbuvir/velpatasvir in people with chronic hepatitis C virus infection and recent injecting drug use: the SIMPLIFY study. Journal of hepatology. 2017; 66: S513.

21. Lu Y, Jin X, Duan CA, et al. Cost-effectiveness of daclatasvir plus asunaprevir for chronic hepatitis C genotype 1b treatment-naive patients in China. Clinical drug investigation. 2018; 13: e0195117.

22. Wu B, Wang Z, Xie Q. Cost-effectiveness of novel regimens for Chinese patients with chronic hepatitis C. Current medical research and opinion. 2018: 1-21.

23. Chen GF, Wei L, Chen J, et al. Will Sofosbuvir/Ledipasvir (Harvoni) Be Cost-Effective and Affordable for Chinese Patients Infected with Hepatitis C Virus? An Economic Analysis Using Real-World Data. PloS one. 2016; 11: e0155934.

24. Wu B, Li T, Chen H, et al. Cost-effectiveness of nucleoside analog therapy for hepatitis B in China: a Markov analysis. Value in health : the journal of the International Society for Pharmacoeconomics and Outcomes Research. 2010; 13: 592-600.

25. Liu S, Cipriano LE, Holodniy M, et al. New protease inhibitors for the treatment of chronic hepatitis C: a cost-effectiveness analysis. Annals of internal medicine. 2012; 156: 279-90.

26. Salomon JA, Weinstein MC, Hammitt JK, et al. Cost-effectiveness of treatment for chronic hepatitis C infection in an evolving patient population. Jama. 2003; 290: 228-37.

27. Thein HH, Krahn M, Kaldor JM, et al. Estimation of utilities for chronic hepatitis C from SF-36 scores. The American journal of gastroenterology. 2005; 100: 643-51.

28. Hagan LM, Yang Z, Ehteshami M, et al. All-oral, interferon-free treatment for chronic hepatitis C: cost-effectiveness analyses. Journal of viral hepatitis. 2013; 20: 847-57.
